# Lower Limb Muscle Strength and Muscle Mass Are Associated With Incident Symptomatic Knee Osteoarthritis: A Longitudinal Cohort Study

**DOI:** 10.3389/fendo.2021.804560

**Published:** 2021-12-16

**Authors:** Nicola Veronese, Sinisa Stefanac, Ai Koyanagi, Nasser M. Al-Daghri, Shaun Sabico, Cyrus Cooper, Renè Rizzoli, Jean-Yves Reginster, Mario Barbagallo, Ligia J. Dominguez, Lee Smith, Stefania Maggi

**Affiliations:** ^1^ Department of Internal Medicine, Geriatric Section, University of Palermo, Palermo, Italy; ^2^ Biochemistry Department, College of Science, King Saud University, Riyadh, Saudi Arabia; ^3^ Institute of Outcomes Research, Centre for Medical Statistics, Informatics and Intelligent Systems, Medical University of Vienna, Vienna, Austria; ^4^ Research and Development Unit, Parc Sanitari Sant Joan de Déu, Universitat de Barcelona, Fundació Sant Joan de Déu, Barcelona, Spain; ^5^ Instituto de Salud Carlos III, Centro de Investigación Biomédica en Red de Salud Mental, Centro de Investigación Biomédica en Red de Salud Mental (CIBERSAM), Madrid, Spain; ^6^ Oxford National Institute for Health Research (NIHR) Musculoskeletal Biomedical Research Unit, Nuffield Department of Orthopaedics, Rheumatology and Musculoskeletal Sciences, Nuffield Orthopaedic Centre, University of Oxford, Oxford, United Kingdom; ^7^ Medical Research Council (MRC) Lifecourse Epidemiology Unit, Southampton General Hospital, University of Southampton, Southampton, United Kingdom; ^8^ National Institute for Health Research Nutrition Biomedical Research Centre, Southampton General Hospital, University of Southampton and University Hospital Southampton National Health System (NHS) Foundation Trust, Southampton, United Kingdom; ^9^ Division of Bone Diseases, Department of Internal Medicine Specialties, Geneva University Hospitals and Faculty of Medicine, Geneva, Switzerland; ^10^ Department of Public Health, Epidemiology and Health Economics, CHU Sart Tilman B23, University of Liege, Liège, Belgium; ^11^ The Cambridge Centre for Sport and Exercise Sciences, Department of Life Sciences, Anglia Ruskin University, Cambridge, United Kingdom; ^12^ National Research Council, Neuroscience Institute, Aging Branch, Padova, Italy

**Keywords:** sarcopenia, osteoarthritis of the knee, older people, epidemiology, muscle mass and function

## Abstract

Recent literature suggests that sarcopenia, often represented by low lower limbs muscle mass and strength, can be considered a potential risk factor for knee osteoarthritis (OA), but the available literature is still limited. We therefore aimed to investigate whether sarcopenia is associated with a higher risk of radiographic (ROA) and symptomatic knee OA (SxOA) in a large cohort of North American people in the context of the OA initiative. Sarcopenia at baseline was diagnosed in case of low skeletal muscle mass (i.e., lower skeletal mass index) and poor performance in the chair stands test. The outcomes of interest for this study included ROA (radiographical osteoarthritis) if a knee developed a Kellgren and Lawrence (KL) grade ≥2 at follow-up, and SxOA (symptomatic osteoarthritis) defined as new onset of a combination of painful knee OA. Altogether, 2,492 older participants (mean age: 68.4 years, 61.4% females) were included. At baseline, sarcopenia was present in 6.1% of the population. No significant difference in ROA prevalence was observed between those with and without sarcopenia (p=0.76), whilst people with sarcopenia reported a significant higher prevalence of SxOA (p<0.0001). Using a logistic regression analysis, adjusting for potential confounders at baseline and the diagnosis of sarcopenia during follow-up, sarcopenia was associated with a higher incidence of knee SxOA (odds ratio, OR=2.29; 95%CI [confidence interval]: 1.42-3.71; p=0.001), but not knee ROA (OR=1.48; 95%CI: 0.53-4.10; p=0.45). In conclusion, sarcopenia could be associated with a higher risk of negative knee OA outcomes, in particular symptomatic forms.

## Introduction

Osteoarthritis (OA) is the most prevalent type of arthritis (1) and a very common long-term disabling chronic condition (2) characterized by the deterioration of cartilage in the joints (3). Evidence suggests that OA is the leading cause of disability worldwide with very high personal, social and economic burdens (4). The prevalence of OA increases with age and is more common in women, people with obesity and those with joint trauma (5). The most prevalent musculoskeletal disease in older adults is knee OA which affects at least 19% of American adults aged 45 years or older (6). Knee OA is characterized by symptomatic and/or radiographic evidence, such as increased pain, functional/joint instability, as well as increased risk of muscle loss and muscle weakness (3). Deterioration of muscle quality and quantity have been linked to sarcopenia (7, 8), thus putting the adults with knee OA at high risk of developing this condition (9).

Based on the latest revised European consensus on definition and diagnosis (2, 10), sarcopenia is a generalized and progressive muscle disorder with an increased likelihood of a variety of poor health outcomes such as falls and fractures (11–13), impaired mobility (14) and gradual loss of independence to perform activities of everyday living (15), eventually leading to respiratory and cardiac diseases (16, 17), low quality of life (QoL) (18) and premature death (19, 20). Moreover, sarcopenia is now recognized to begin earlier in life and is not merely related to ageing as previously presumed (21).

Even though sarcopenia often accompanies OA (22), the association between them is still unclear and due to the conflicting results and insufficient evidence, no agreement has been reached (23, 24). Considering the increasing evidence of negative health outcomes that are associated with these two conditions (25), we aimed to investigate whether sarcopenia is associated with a higher risk of radiographic (ROA) and symptomatic knee OA (SxOA) in a large cohort of North American people followed-up for 4 years.

## Materials and Methods

### Data Source and Subjects

Data for this study were obtained from the Osteoarthritis Initiative (OAI) database (https://nda.nih.gov/oai/). In the OAI, participants were recruited across four clinical sites in the United States of America (Baltimore, MD; Pittsburgh, PA; Pawtucket, RI; and Columbus, OH) between February 2004 and May 2006. In the OAI project, individuals were included if they: (1) had knee OA with knee pain for a 30-day period in the past 12 months or (2) were at high risk of developing knee OA (e.g. obese/overweight, family traits for knee OA) (26). For the aims of this work, the data were collected at baseline, in the screening evaluations and in subsequent evaluations until four years of follow-up.

All participants provided written informed consent. The OAI study was given full ethics approval by the institutional review board of the OAI Coordinating Center, at the University of California in San Francisco.

### Sarcopenia Definition (Exposure)

For the definition of sarcopenia, we used the criteria of the revised European consensus on the definition and diagnosis of sarcopenia (10). Sarcopenia was defined as a chair stand test time >15 seconds for 5 repetitions (muscle strength parameter) and low skeletal muscle mass (SMM) as reflected by lower skeletal mass index (SMI) (body composition parameter) (10).

SMM was calculated based on the equation proposed by Lee and colleagues (27): SMM= 0.244*weight + 7.8*height + 6.6*sex – 0.098*age + race – 3.3 [where female=0 and male=1; race=0 (White and Hispanic), race=1.9 (Black) and race=-1.6 (Asian)]. SMM was further divided by body mass index (BMI) based on weight and height measured by a trained nurse, to create a SMI (28). Low SMM was defined as the lowest quartile of the SMI based on sex-stratified values (29).

### Assessment of Knee OA Outcomes

At baseline and during follow-up examinations, individuals had full knee assessments which included both clinical and radiographic examinations. A fixed flexion posterior–anterior radiograph, which was read centrally for Kellgren and Lawrence (KL) grade, was made for all the participants. In addition, participants were asked regarding knee pain, the following question: ‘During the past 30 days, have you had pain, aching, or stiffness in your right/left knee on most days?’.

The outcomes of interest for this study included: (1) ROA (radiographical osteoarthritis) if a knee developed a KL grade ≥2 at follow-up among those without this condition at baseline and (2) SxOA (symptomatic osteoarthritis), defined as the presence of a combination of painful knee OA. The assessment of the knee OA outcomes was made, other than at baseline, at V01 (12 months), V03 (24 months), V05 (36 months), and V06 (48 months).

### Covariates

Several covariates at baseline other than age and sex were identified as potential confounding factors based on previous literature (30). These included: race (whites vs. others); educational attainment (college or higher vs. others); yearly income (< vs ≥ $50,000 or missing data); smoking habits; physical activity evaluated using the total score for the Physical Activity Scale for the Elderly scale (PASE) (31); Charlson Comorbidity Index score (32), a validated general health measure of self-reported comorbidities; the number of medications used; daily energy intake (in Kcal).

### Statistical Analyses

Data on continuous variables were normally distributed according to the Kolmogorov-Smirnov test. Data were consequently presented as means and standard deviation values (SD) for quantitative measures, and percentages for all categorical variables by the presence or absence of sarcopenia at baseline. P values were calculated using an independent T test for continuous variables and a chi-square test for categorical parameters.

To assess the association between sarcopenia and the outcomes of interest during follow-up, a logistic regression analysis was applied, since a survival analysis was not possible due to lack of information on the precise date of event. The basic adjusted model included age and gender. The fully adjusted model included all parameters associated with the outcomes of interest (p-value <0.10) or significantly different between sarcopenic and non-sarcopenic subjects (p-value <0.05). Multi-collinearity among covariates was assessed through variance inflation factor (VIF) (33), taking a cut-off of 2 as the criterion for exclusion. No covariates were excluded using this criterion. Adjusted odds ratios (OR) and 95% confidence intervals (CI) were calculated to estimate the strength of the associations between sarcopenia at baseline and incident knee OA outcomes.

A p<0.05 was deemed statistically significant. All analyses were performed using SPSS^®^ software version 21.0 for Windows (SPSS Inc., Chicago, Illinois).

## Results

### Sample Selection

**Figure 1:**
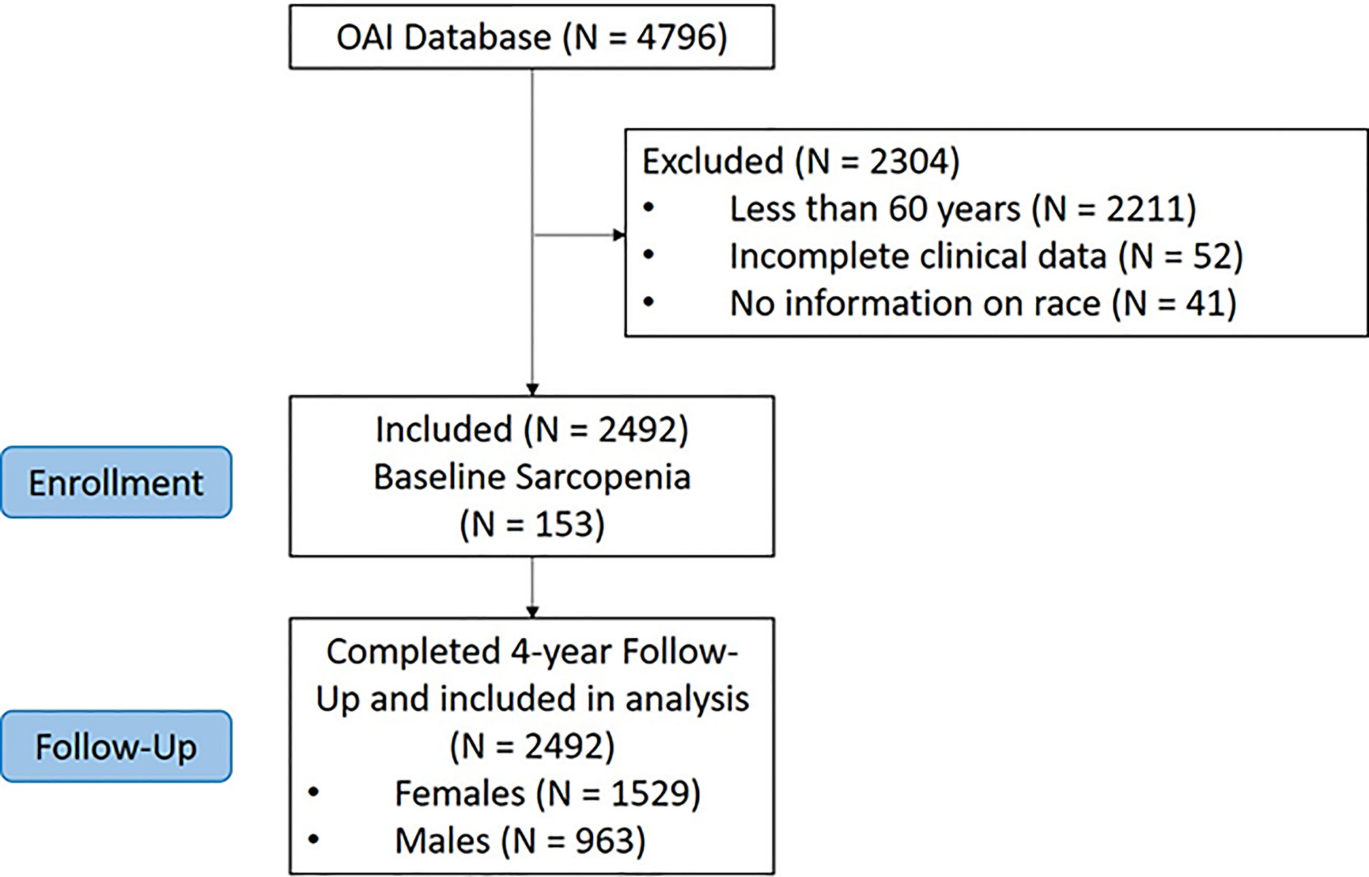
Flow-chart of the participants included.

The OAI database initially included a total of 4,796 participants. We excluded 2,211 individuals since they were less than 60 years of age, 52 since no data regarding body composition or chair stands time were available, and 41 for not having data regarding race. Therefore, 2,492 participants were included, as shown in **Figure 1**.

### Descriptive Characteristics

The cohort consisted of 1,529 females (61.4%), with a mean age of 68.4 years ( ± 5.4 years; range: 60-79 years). The prevalence of sarcopenia at baseline was 6.1%, affecting 153 subjects. At baseline, 58% were affected by knee ROA and, of them, 24.4% knee SxOA.

**Table 1** shows the baseline characteristics by the presence of sarcopenia. Compared to the 2,239 participants without sarcopenia, sarcopenic subjects were significantly older, more sedentary, with a lower educational level and were poorer (**Table 1**). Sarcopenic individuals had a greater number of comorbidities, and they used a higher number of medications. Finally, no significant difference in ROA prevalence was observed between sarcopenic and non-sarcopenic individuals (p=0.76), whilst people with sarcopenia reported a significantly higher prevalence of SxOA (69.4% vs. 49.5%; p<0.0001) than their counterparts (**Table 1**).

**Table 1:** Baseline characteristics of participants according to the presence of sarcopenia.

	Sarcopenia (n=153)	No sarcopenia (n=2239)	p-value
***General characteristics***			
**Age (years)**	72.1 (5.3)	68.2 (5.4)	<0.0001
**Males (%)**	41.2	38.5	0.55
**PASE (points)**	114 (56)	139 (67)	<0.0001
**Whites (%)**	88.2	83.9	0.17
**Smoking (previous/current) (%)**	43.1	51.1	0.07
**Graduate degree (%)**	20.3	29.0	0.02
**Yearly income (≥ $50,000) (%)**	36.3	54.8	<0.0001
**Daily energy intake (Kcal)**	1334 (565)	1334 (538)	0.99
***Medical conditions***			
**BMI (kg/m^2^)**	30.8 (5.0)	28.2 (4.5)	<0.0001
**Charlson co-morbidity index (points)**	0.79 (1.10)	0.45 (0.88)	<0.0001
**Number of medications**	4.14 (2.97)	3.37 (2.67)	0.001
***Osteoarthritis items***			
**ROA (%)**	9.1	10.0	0.76
**SxOA (%)**	69.4	49.5	<0.0001

The data are presented as mean (standard deviation) for continuous variables and percentages (%) for categorical outcomes.

CES-D, Center for Epidemiologic Studies Depression Scale; PASE, Physical Activity Scale for the Elderly; BMI, body mass index; OA, osteoarthritis; ROA, radiographic OA; SxOA, symptomatic knee OA.

### Sarcopenia and Incident Knee Osteoarthritis Outcomes

During the four years of follow-up, the incidence of ROA was 10.7% and that of SxOA 46.9%. As shown in **Table 2**, using a logistic regression analysis, adjusting for potential confounders at baseline and the diagnosis of sarcopenia during follow-up, sarcopenia was associated with a higher incidence of knee SxOA (OR=2.29; 95%CI: 1.42-3.71; p=0.001), but not knee ROA (OR=1.48; 95%CI: 0.53-4.10; p=0.45).

**Table 2:** Association between baseline sarcopenia and incident knee OA outcomes.

	N	Basic adjusted model (OR, 95%CI)	p-value	Fully adjusted model^1^ (OR, 95%CI)	p-value
**ROA**	847	1.38 (0.51-3.76)	0.52	1.48 (0.53-4.10)	0.45
**SxOA**	1683	2.51 (1.56-4.02)	<0.001	2.29 (1.42-3.71)	0.001

All the data are presented as odds ratios (95% confidence intervals).

^1^ Basic adjusted model included as covariates age (as continuous) and sex;

^2^ Fully adjusted model included as covariates, other age and sex: race (whites vs. others); education (degree vs. others); yearly income (categorized as ≥ or < 50,000$ or missing data); smoking habits (current and previous vs. others); Physical Activity Scale for Elderly score (as continuous); Charlson co-morbidity index; number of medications used; energy intake (as continuous).

CI, confidence intervals; OR, odds ratio; ROA, radiographic OA; SxOA, symptomatic knee OA.

## Discussion

In this research involving 2,492 older people, we found that the presence of sarcopenia at baseline was significantly associated with a higher risk of symptomatic knee OA, over four years of follow-up.

The overall prevalence of sarcopenia at baseline in our study was just under 10%, which is in accordance to the latest systematic review and meta-analysis of general population studies (34). The existing literature supports our finding of sarcopenia in older people (35, 36) as well as those with lower educational level and lower income (37), more comorbidities, higher medication intake and more sedentary lifestyle (38, 39).

Other works already explored the potential association between sarcopenia and knee OA outcomes in older people. Recently, Andrews et al. reported that sarcopenia could be associated with a higher risk of sarcopenia, in Health, Aging, and Body Composition participants (40). Interestingly, our findings did not show any significant difference between ROA prevalence and people with or without sarcopenia, according to the paper of Andrews et al. (40). However, a significantly higher prevalence of symptomatic knee OA was reported. In another study, whose aim was to explore the prevalence and characteristics of pain associated with sarcopenia, it was found that the prevalence of pain was much higher in participants with sarcopenia than their counterparts (41). These findings overall suggest that sarcopenia could be associated with higher risk of pain associated to knee OA, indicating the need of early identification of these patients for tailored interventions.

For example, physical exercise interventions could be suggested in people with sarcopenia and without symptomatic knee OA since this kind of intervention is able to prevent further muscle mass loss (42), incident knee OA (in particular forms associated to pain) (43), and pain itself (44). In this regard, it should be acknowledged that muscle strengthening exercises can improve physical performance and muscle strength parameters in people affected by sarcopenia (45), having the potential to probably prevent symptomatic knee OA.

The strengths of our study are the long duration of follow-up, the several knee OA outcomes assessed, and the large sample size included. However, our findings should be interpreted within some important limitations. First, the participants of the OAI were at high risk or already had knee OA. Thus, our results cannot be extended to the general population. Second, the observational nature of our findings can introduce another bias in our results, although we tried to correct this limitation using analyses adjusted for potential confounders. Finally, body composition was based on a population equation and not on direct assessment. However, this has been validated against gold standard methods such as magnetic resonance imaging and dual-energy X-ray absorptiometry (27). At the same time, sarcopenia was identified using lower limbs performance and muscle mass, whilst handgrip strength is the preferred method for diagnosis sarcopenia (10).

In conclusion, our study suggests that sarcopenia could be associated with a higher risk of negative knee OA outcomes and in particular symptomatic forms. Our findings further suggest the importance of early detection of sarcopenia, in order to implement appropriate preventive treatment against the progression of knee OA.

## Data Availability Statement

The dataset supporting the conclusions of this article is freely available in https://nda.nih.gov/oai/.

## Ethics Statement

The OAI study was given full ethics approval by the institutional review board of the OAI Coordinating Center, at the University of California in San Francisco. The patients/participants provided their written informed consent to participate in this study.

## Author Contributions

Manuscript preparation, NV, SiS, NA-D, and ShS. Critical revision, SM, LS, CC, RR, and J-YR. Data interpretation, MB, and LD. Statistical analysis, NV. All authors contributed to the article and approved the submitted version.

## Funding

The authors thank the support of the Researchers Supporting Project (RSP-2021/21) King Saud University, Riyadh, Saudi Arabia. The OAI is a public-private partnership comprised of five contracts (N01-AR-2-2258, N01-AR-2-2259, N01-AR-2-2260, N01-AR-2-2261, and N01-AR-2-2262) funded by the National Institutes of Health, a branch of the Department of Health and Human Services, and conducted by the OAI Study Investigators. Private funding partners include Merck Research Laboratories, Novartis Pharmaceuticals Corporation, GlaxoSmithKline, and Pfizer, Inc. Private sector funding for the OAI is managed by the Foundation for the National Institutes of Health. This manuscript was prepared using an OAI public use data set and does not necessarily reflect the opinions or views of the OAI investigators, the NIH, or the private funding partners.

## Conflict of Interest

The authors declare that the research was conducted in the absence of any commercial or financial relationships that could be construed as a potential conflict of interest.

## Publisher’s Note

All claims expressed in this article are solely those of the authors and do not necessarily represent those of their affiliated organizations, or those of the publisher, the editors and the reviewers. Any product that may be evaluated in this article, or claim that may be made by its manufacturer, is not guaranteed or endorsed by the publisher.
